# A rare case report of polyangiitis overlap syndrome: granulomatosis with polyangiitis and eosinophilic granulomatosis with polyangiitis

**DOI:** 10.1186/s12890-018-0733-2

**Published:** 2018-11-29

**Authors:** Michele V. Quan, Stephen K. Frankel, Mehrnaz Maleki-Fischbach, Laren D. Tan

**Affiliations:** 1 Department of Pulmonary, Critical Care, Hyperbaric and Sleep Medicine, Loma Linda University Medical Center, 11234 Anderson St., Loma Linda, CA 92354 USA; 2 Division of Pulmonary, Critical Care and Sleep Medicine, National Jewish Health, 1400 Jackson St., Denver, CO 80206 USA; 3 Division of Rheumatology, National Jewish Health, 1400 Jackson St., Denver, CO 80206 USA

**Keywords:** Granulomatosis with polyangiitis, Wegener’s, GPA, Eosinophilic granulomatosis with polyangiitis, Churg-Strauss, EGPA, Overlap syndrome, Wegener’s with eosinophilia, GPA with eosinophilia

## Abstract

**Background:**

Granulomatosis with polyangiitis (GPA) is a systemic ANCA-associated vasculitis characterized by necrotizing granulomatous inflammation and a predilection for the upper and lower respiratory tract. Eosinophilic granulomatosis with polyangiitis (EGPA) is also a systemic ANCA-associated vasculitis, but EGPA is characterized by eosinophilic as well as granulomatous inflammation and is more commonly associated with asthma and eosinophilia.

Polyangiitis overlap syndrome is defined as systemic vasculitis that does not fit precisely into a single category of classical vasculitis classification and/or overlaps with more than one category. Several polyangiitis overlap syndromes have been identified, however, there are very few case reports of an overlap syndrome involving both GPA and EGPA in the medical literature.

**Case presentation:**

We conducted a PUBMED literature review using key words ‘granulomatosis with polyangiitis,’ ‘Wegener’s,’ ‘GPA,’ ‘eosinophilic granulomatosis with polyangiitis,’ ‘Churg-Strauss,’ ‘EGPA,’ ‘overlap syndrome,’ ‘Wegener’s with eosinophilia,’ and ‘GPA with eosinophilia’ in English only journals from 1986 to 2017. Relevant case reports and review articles of overlap syndromes of GPA and EGPA were identified. We aim to report a unique case of GPA and EGPA overlap syndrome and review the cases that have been previously described.

Between 1986 and 2017, we identified 15 cases that represent an overlap syndrome with compelling features of both GPA and EGPA. Patients ranged in age between 21 and 78. Of those whose gender was identified, 80 % of the patients were female. All cases described involved the lungs, 60 % reported sinus involvement, and more than 50 % displayed renal involvement. An overwhelming majority of patients were positive for c-ANCA and demonstrated eosinophilia (peripheral blood or tissue eosinophilia). A preponderance of the cases described were treated with systemic corticosteroids combined with an immunosuppressive/cytotoxic agents.

**Conclusion:**

To our knowledge, there have been very few cases reported of an overlap syndrome of GPA and EGPA. Identification of patients with a polyangiitis overlap syndrome of GPA and EGPA is imperative as prognosis, longitudinal management and treatment modalities may differ between these entities.

## Background

Vasculitis encompasses a group of clinical entities, all of which share the histopathologic hallmark of inflammation of blood vessel walls. One classification scheme categorizes the vasculitides by the size of the affected vessels, and in this paradigm, “small vessel vasculitis” predominantly affects the small intraparenchymal arteries, arterioles, capillaries, and venules [1]. Anti-neutrophil cytoplasmic antibody (ANCA)-associated vasculitis is a small vessel vasculitis in which very few or no immune deposits are found in the vessel wall but is commonly associated with circulating anti-neutrophil cytoplasmic antibodies. The specific entities of granulomatosis with polyangiitis (GPA) (formerly known as Wegener granulomatosis), microscopic polyangiitis, and eosinophilic granulomatosis with polyangiitis (EGPA) (formerly known as Churg Strauss Syndrome) compose the ANCA associated vasculitides.

Granulomatosis with polyangiitis is characterized by necrotizing granulomatous inflammation that usually involves the upper and lower respiratory tracts as well as by necrotizing, crescentic glomerulonephritis. Constitutional symptoms, destructive sinus or otic disease, ocular involvement, cutaneous involvement, neurologic involvement, musculoskeletal manifestations, and pulmonary capillaritis with hemorrhage are frequently encountered [2, 3]. Still, end-organ manifestations vary from patient to patient, and disease severity may vary from limited disease (upper airway, ocular or isolated pulmonary manifestations) to generalized disease to life-threatening disease (alveolar hemorrhage, cardiac or central nervous system involvement) [4]. GPA is associated with ANCA (primarily c-ANCA) and anti-proteinase-3 antibody (anti-PR3) positivity. Ultimately, the diagnosis of GPA, or any vasculitis, requires that the clinician fully integrate the clinical, radiologic, laboratory and histologic information and findings and reach a conclusion that the preponderance of the data does or does not support a compelling diagnosis of GPA.

Eosinophilic granulomatosis with polyangiitis is classically described as presenting with the triad of necrotizing vasculitis, asthma, and eosinophilia. Asthma is nearly universal, and while it is commonly severe and of long duration, it is not necessarily so. Cardiac involvement is relatively common (on the order of 30–50%) and accounts for a significant percentage of the attributable morbidity and mortality of EGPA. Other common target end organs include the skin, peripheral nervous system, pulmonary parenchyma (eosinophilic pneumonia and/or radiographic infiltrates), sinuses, musculoskeletal system, gastrointestinal tract and kidneys. Approximately one-half to three-quarters of patients will be either ANCA (primarily p-ANCA) or anti-myeloperoxidase (MPO) positive. Limited presentations of EGPA may occur as well [5].

Overlap syndrome was postulated in 1954 by Godman and Churg, who described a patient with Wegener granulomatosis and eosinophilic pulmonary infiltrates [6]. Polyangiitis overlap syndrome, previously published by Leavitt and Fauci, is defined as systemic vasculitis that does not fit precisely into a single category of classical vasculitis or overlaps more than one subtype of vasculitis [7]. Several polyangiitis overlap syndromes have been identified, however, to our knowledge, there are only several case reports of an overlap syndrome involving both GPA and EGPA [8–16].

We conducted a PUBMED literature review using key words ‘granulomatosis with polyangiitis,’ ‘Wegener’s,’ ‘GPA,’ ‘eosinophilic granulomatosis with polyangiitis,’ ‘Churg-Strauss,’ ‘EGPA,’ ‘overlap syndrome’ ‘Wegener’s with eosinophilia’ and ‘GPA with eosinophilia’ in English only journals from 1986 to 2017. Relevant case reports and review articles were found that described cases of overlap syndromes of GPA and EGPA. In this manuscript we report a unique case of GPA/EGPA overlap syndrome and place this case in the context of the other cases that have been previously described.

## Case presentation

**Fig. 1:**
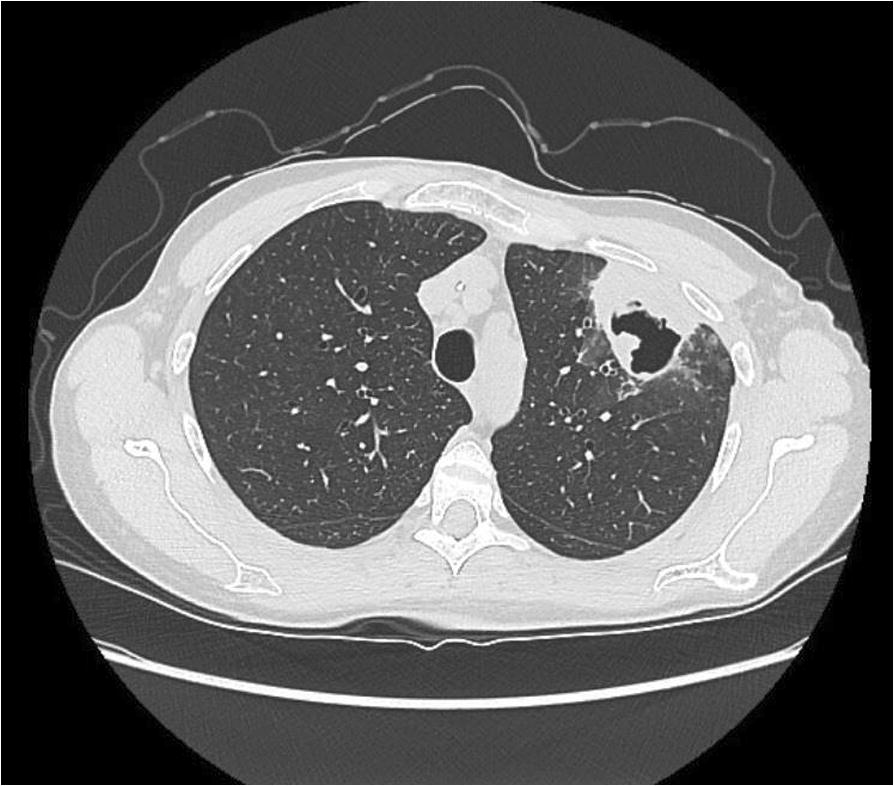
CT imaging revealing left upper lobe consolidation with central cavitary lesion, adjacent scattered consolidation, ground-glass opacities and tree-in-bud markings

A 50-year-old woman presented with an episode of hemoptysis. She also reported low grade fevers, night sweats and chest discomfort. Her past medical history was notable for allergic rhinitis/sinusitis and asthma, and she experienced recurring upper respiratory tract infections, sinusitis, and bronchitis approximately three times per year. Chest computed tomography imaging revealed left upper lobe consolidation with a central cavitary lesion, adjacent scattered consolidation, ground-glass opacities and tree-in-bud markings (Fig. 1). She subsequently underwent bronchoscopy, and bronchoalveolar lavage grew Mycobacterium avium complex (MAC). Consequently, she underwent cavitary MAC treatment with rifampin, ethambutol, erythromycin, and intravenous amikacin with improvements in low-grade fever, night sweats, and chest discomfort.

Four months later, her symptoms returned, and chest imaging revealed a new right basilar lung infiltrate, with increased ground-glass and consolidative focal patchy disease, as well as bronchiectasis. Repeat bronchoscopy revealed a positive fungal culture for Aspergillus and she was subsequently started on voriconazole. Upon re-evaluation, the patient exhibited oral ulcers, purpuric skin lesions, and new onset peripheral neuropathy and was referred for an additional opinion.

Pertinent laboratory findings included a complete blood count with a total white cell count of 11,000 cells per microliter with marked hypereosinophilia at 5200 eosinophils per microliter. Erythrocyte sedimentation rate was elevated at 92 mm/hr., and C-reactive protein was elevated at 1.154 mg/dL (normal less than 0.4). Quantitative serum immunoglobulin E (IgE) was also elevated at 520 international units/mL (normal 0 to 11). Patient was negative for ANCA, hepatitis B or C, PR3 antibody and MPO antibody.

Pulmonary function testing demonstrated normal total lung capacity (TLC) and thoracic gas volume but an elevated residual volume at 2.93 L (154% of predicted) suggesting mild air trapping. Airflows were normal, with a forced expiratory volume in one second (FEV1) of 2.67 L (90% of predicted) and forced vital capacity (FVC) was 3.23 L (86% of predicted) with no significant bronchodilator response. Diffusing capacity of the lungs for carbon monoxide (DLCO) was mildly reduced at 20.97 or 77% of predicted, but when corrected for alveolar volume (VA), corrected to normal with a DLCO/VA of 4.59 or 86% of predicted. The VA was significantly less than the TLC, suggesting gas mal-distribution.

**Fig. 2:**
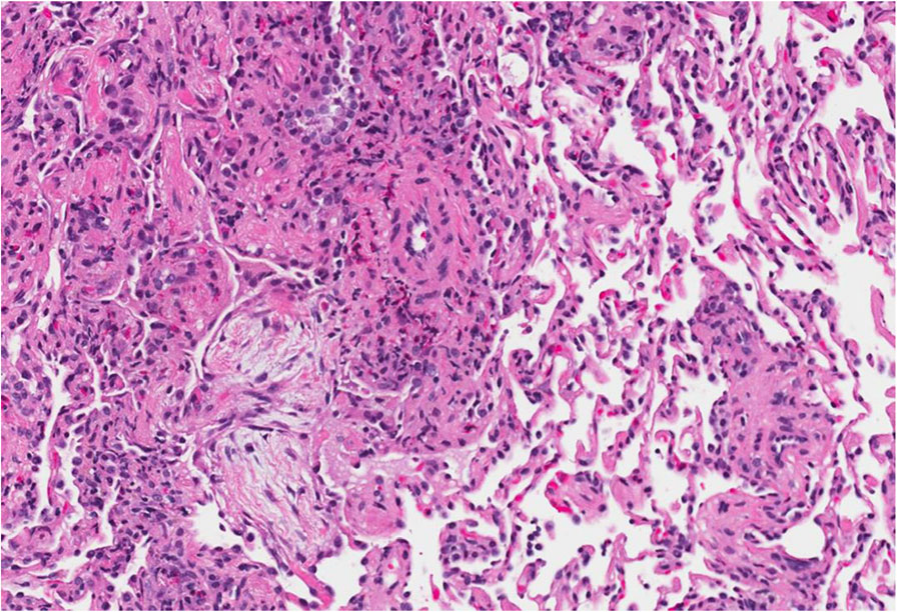
20X field of an H&E stain (hematoxylin and eosin stain) with perivascular eosinophils and a neighboring airspace with a plug of organizing pneumonia

**Fig. 3:**
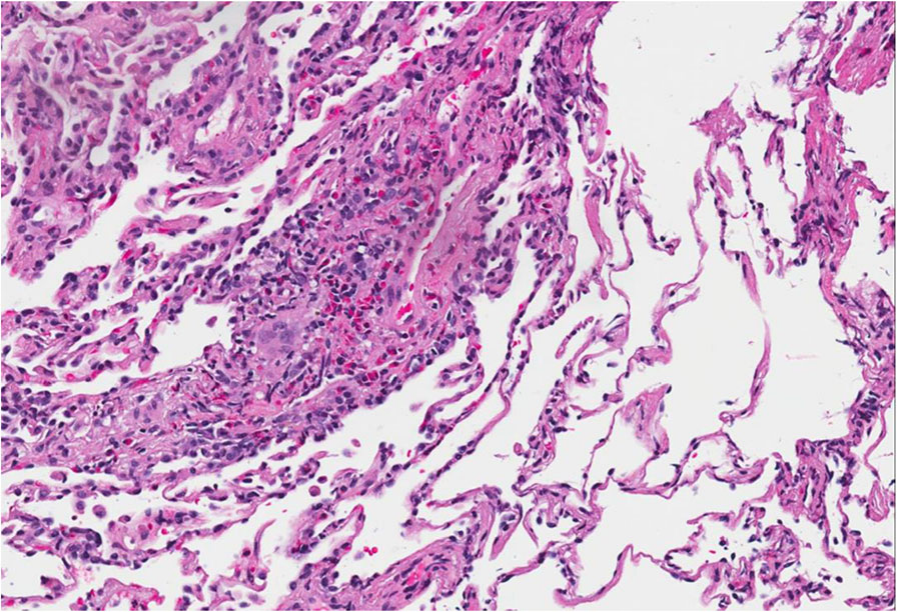
20X field of H&E stain with perivascular eosinophils

**Fig. 4:**
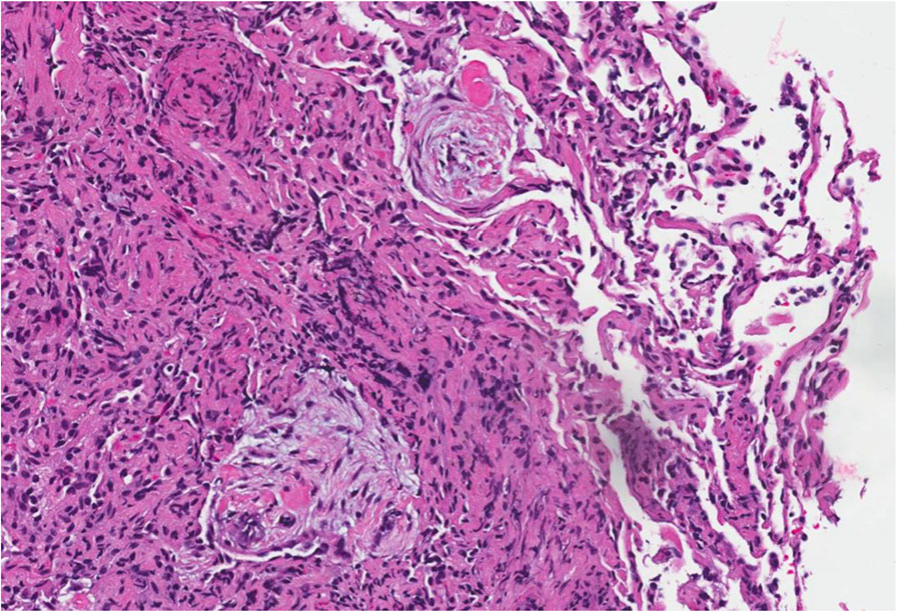
20X field of H&E stain with plugs of organizing pneumonia and fibrin

Repeat bronchoscopy was performed with transbronchial biopsies. Histopathology showed acute and chronic inflammation, scattered eosinophils (42 eosinophils per 60 × high power field), fresh hemorrhage and tissue eosinophilia most prominently in the bronchial wall and adjacent alveolated lung parenchyma. There was no evidence of granuloma formation or necrotizing vasculitis (Figs. 2, 3 and 4).

The constellation of compelling clinical, radiographic, laboratory, and pathologic findings believed to suggest GPA/EGPA overlap syndrome included a cavitary pulmonary lesion unresponsive to anti-microbial therapy, oral ulcers, purpuric skin lesions, peripheral neuropathy, long-standing asthma, chronic sinusitis, one or more episodes of alveolar hemorrhage, migratory pulmonary infiltrates on chest imaging, peripheral hypereosinophilia, a markedly elevated IgE level, elevated inflammatory markers and eosinophilic inflammation in the pulmonary parenchyma on histopathologic examination.

In addition to the very complex clinical picture most suggestive of EGPA/GPA overlap syndrome, there was compelling evidence for a concomitant aspergillus pulmonary infection, and the patient also met criteria for a diagnosis of allergic bronchopulmonary aspergillosis. There is a possibility that the aspergillus pulmonary infection may have complicated our patient’s clinical picture as it may have contributed to her pulmonary cavitation and sinusitis.

While the patient had been previously treated with oral corticosteroids and anti-fungal therapy for a prolonged period of time with little improvement, with the addition of anti-interleukin (IL)-5 therapy and cytotoxic therapy (azathioprine) to the oral corticosteroids and anti-fungal therapy, the patient had dramatic improvement in her disease manifestations.

Between 1986 and 2017, we identified 15 cases that described an overlap syndrome involving both GPA and EGPA (Table 1). Patients ranged in age between 21 and 78. Of those whose sexes were identified, 80 % of cases described were female. All cases described involved the lungs, 60 % described sinus involvement, and over half had displayed renal involvement. An overwhelming majority of patients were positive for c-ANCA and demonstrated eosinophilia (peripheral or tissue involvement). A preponderance of the cases described were treated with systemic corticosteroids combined with cytotoxic/immunosuppressive agents.

**Table 1:** Clinical features of cases that describe EGPA and GPA overlap syndrome

Case	Age/Sex	Involved Organs	h/o Asthma	Eosinophilia	ANCA	Treatment
Henochowichz 1986 [24]	25 F	J, K, L (AH), N, Sk	no	P: yes, K/N: yes	N/A	Cs, CTX
Yousem 1989 [10]	71 F	E, L, N, S	yes	P: no, L: yes	N/A	Cs, AZA
Krupsky 1993 [16]	43 M	J, L, Ne, S, Sk	no	P: yes, L: yes	+cANCA	Cs, CTX
Potter 1999 [25]- Case 1	29 M	J, L (AH), N, S	no	P: yes, L: yes	+cANCA, +PR3, neg pANCA	Cs, MTX, TMP-SMX
Potter 1999 [25]- Case 2	39 F	J, L (AH), S	no	P: yes, L: yes	+cANCA, +PR3, neg pANCA	Cs, MTX, CTX, TMP-SMX
Lane 2002 [14]- Case 1	N/A	L, K	N/A	K: yes	+cANCA	N/A
Lane 2002 [14]- Case 2	N/A	L, K, N, S	N/A	K: yes	+cANCA, +PR3	N/A
Lane 2002 [14]- Case 3	N/A	L, K, N, S	N/A	P: yes, K: yes	+cANCA	N/A
Lane 2002 [14]- Case 4	N/A	L, K	N/A	P: yes, K: yes	+cANCA, +PR3	N/A
Lane 2002 [14]- Case 5	N/A	L, K	N/A	K: yes	+cANCA	N/A
Kamali 2003 [15]	21 F	J, K, L (AH), Sk	no	P: yes	+cANCA, +PR3	Cs, CTX, IVIG
Shoda 2005 [13]	25 F	E, J, L (AH), N, S, Sk	no	P: yes, L/N: yes	+cANCA, +PR3, neg pANCA	Cs, MTX, CTX
Uematsu 2014 [8]	78 F	L (AH), S, Sk	no	P: yes	+cANCA, +PR3, neg pANCA	Cs, CTX
Surendran 2017 [9]	45 F	E, J, L (AH), K, N, Ne	yes	P: no	+cANCA, +PR3	Cs, CTX
Our Patient 2017	50 F	L (AH), Ne, S	yes	P: yes, L: yes	neg	Cs, AZA, IL-5

*M* male, *F* female, *N* nose, *E* eye, *S* sinus, *Sk* skin, *J* joint, *L* lung, *AH* alveolar hemorrhage, *Ne* neuropathy, *K* kidney, *P* peripheral, *PR3* proteinase-3 antibody, *Cs* corticosteroids, *AZA* azathioprine, *CTX* cyclophosphamide, *MTX* methotrexate, *TMP-SMX* trimetophrim-sulfamethoxazole, *IL-5* anti-interleukin-5 therapy

## Discussion and conclusions

Several polyangiitis overlap syndromes have been identified, however, to our knowledge, there are few reports of an overlap syndrome involving both GPA and EGPA. It is essential to identify patients with GPA/EGPA overlap syndrome because treatment modalities differ for GPA and EGPA.

Uematsu et al. diagnosed overlap syndrome with GPA and EGPA in a patient because of the combination of peripheral eosinophilia and asthmatic symptoms with an elevated serum PR3-ANCA level and negative MPO-ANCA titers [8]. Skin biopsy revealed focal panarteritis with necrotizing vasculitis. Additionally, the patient developed nasal obstruction, epistaxis and microscopic hematuria, which are much more commonly seen in patients with GPA than in patients with EGPA.

Surendran et al. identified overlap syndrome in a patient with similar features including the presence of polyarthritis, pan-sinusitis, c-ANCA positivity, rapidly progressive glomerulonephritis, and mononeuritis multiplex arguing for both EGPA and GPA [9]. The presence of a history of asthma and tissue eosinophilia favored EGPA, while the presence of nasal septal perforation and eye proptosis favored GPA.

The clinical findings most commonly featured throughout the identified overlap syndromes were a constellation of characteristic end organ manifestations in the lungs, kidneys, and sinuses plus eosinophilia (whether peripheral or tissue).

The treatment of both GPA and EGPA depends upon disease severity or risk stratification. As a general rule, therapeutic agents used for the management of GPA and EGPA have a very real risk of adverse effects and must be used with care even as the clinician recognizes the life-threatening nature of the vasculitis itself. For patients with more mild disease, oral glucocorticoids may be sufficient, whereas patients with generalized active disease will usually require therapy with cytotoxic agents such as methotrexate, azathioprine, cyclophosphamide or mycophenolate mofetil [17, 18]. For patients with GPA, rituximab has been shown to be effective in generalized active and severe or life-threatening disease (e.g. alveolar hemorrhage, cardiac involvement, CNS involvement or fulminant renal failure) [20–22]. For patients with EGPA, anti-interleukin-5 monoclonal antibodies have recently been shown to have efficacy. Specifically, mepolizumab, a humanized monoclonal antibody to IL-5, has been evaluated in patients with EGPA in a randomized trial and multiple case reports. In a multicenter trial, 44% of mepolizumab- treated subjects were able to taper glucocorticoids compared with 7 % of subjects taking placebo [19].

**Fig. 5:**
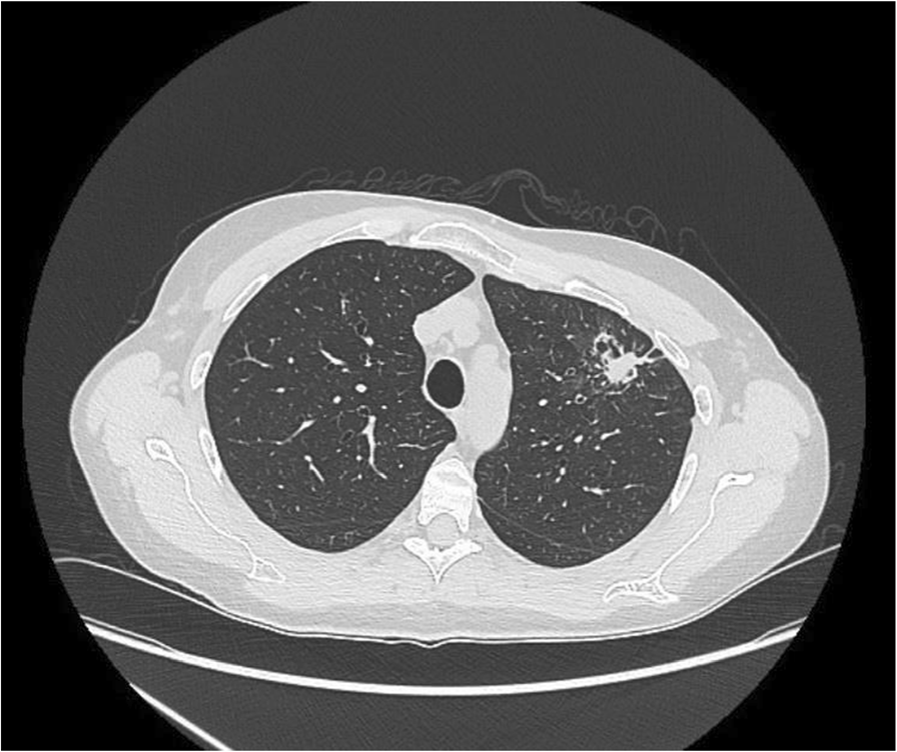
Follow up CT imaging with resolving left upper lobe cavitary lesion after initiation of corticosteroids, azathioprine and anti-IL-5

Review of the available overlap EGPA and GPA literature revealed that the majority of patients were treated with a combination of corticosteroids and immunosuppressive agents. Cyclophosphamide was most commonly used, followed by methotrexate and azathioprine (although to some degree this likely reflects the practice patterns before the introduction of newer agents such as rituximab and mepolizumab). Our patient was treated with a combination of corticosteroids, azathioprine, and anti-IL-5 therapy with marked improvement in her symptoms and follow-up CT scan (Fig. 5).

While our patient did not have evidence of biopsy proven vasculitis on transbronchial biopsy, there is evidence that transbronchial biopsies of alveolar tissue are seldom positive in pulmonary vasculitides [23].

In conclusion, patients may present with defined GPA, defined EGPA or in rare instances an overlap syndrome of EGPA and GPA. While manifestations are variable, the reported cases have presented with a combination of asthma, sinus involvement, polyarthritis, lung involvement, renal involvement, c-ANCA positivity, eosinophilia, and histopathologic vasculitis.

Identification of patients with polyangiitis overlap syndrome of GPA and EGPA is imperative because treatment options differ. Patients with EGPA are able to achieve disease remission with corticosteroids and now anti-IL5 therapy, however, patients with GPA or polyangiitis overlap syndrome may require a combination of corticosteroids, biologic therapy and cytotoxic agents.

## Abbreviations

ANCA: Anti-neutrophil cytoplasmic antibody
DLCO: Diffusing capacity of the lungs for carbon monoxide
EGPA: Eosinophilic granulomatosis with polyangiitis
FEV1: Forced expiratory volume in one second
FVC: Forced vital capacity
GPA: Granulomatosis with polyangiitis
IgE: Quantitative serum immunoglobulin E
IL-5: Anti-interleukin-5
MAC: Mycobacterium avium complex
MPO: Anti-myeloperoxidase
PR3: Anti-proteinase-3 antibody
TLC: Total lung capacity
VA: Alveolar volume
